# Supplementary material for: Risk factors for bronchopulmonary dysplasia in preterm infants: a systematic review and meta-analysis
Source: PeerJ. 2025 Oct 10;13:e20202. doi: 10.7717/peerj.20202 (PMC12517283; doi:10.7717/peerj.20202)
Supplement: Supplemental Information 1 — Detailed search strategy for the systematic review and meta-analysis on risk factors of bronchopulmonary dysplasia, including databases (e.g., PubMed, Embase), search terms (e.g., ’prematurity’, ’ bronchopulmonary dysplasia ’ ,’risk factors’), and Boolean operators (AND/OR). [file peerj-13-20202-s001.docx]

Search strategy

**PubMed**(872) （2024.11.22）

| Search number | Query | Results |
| --- | --- | --- |
| #1 | "Bronchopulmonary Dysplasia"[Mesh] | 6561 |
| #2 | (Dysplasia, Bronchopulmonary[Title/Abstract]) OR (Chronic lung disease of prematurity[Title/Abstract]) | 300 |
| #3=#1OR#2 | ("Bronchopulmonary Dysplasia"[Mesh]) OR ((Dysplasia, Bronchopulmonary[Title/Abstract]) OR (Chronic lung disease of prematurity[Title/Abstract])) | 6740 |
| #4 | "Infant, Premature"[Mesh] | 68214 |
| #5 | (((((((Infants, Premature[Title/Abstract])OR (Premature Infant[Title/Abstract])) OR (Premature Infants[Title/Abstract])) OR (Preterm Infants[Title/Abstract])) OR (Infant, Preterm[Title/Abstract])) OR (Infants, Preterm[Title/Abstract])) OR (Preterm Infant[Title/Abstract])) OR (Neonatal Prematurity[Title/Abstract]) | 54787 |
| #6=#4OR#5 | ("Infant, Premature"[Mesh]) OR ((((((((Infants, Premature[Title/Abstract]) OR (Premature Infant[Title/Abstract])) OR (Premature Infants[Title/Abstract])) OR (Preterm Infants[Title/Abstract])) OR (Infant, Preterm[Title/Abstract])) OR (Infants, Preterm[Title/Abstract])) OR (Preterm Infant[Title/Abstract])) OR (Neonatal Prematurity[Title/Abstract])) | 88242 |
| #7 | "Infant, Newborn"[Mesh] | 696448 |
| #8 | ((((((Infants, Newborn[Title/Abstract]) OR (Newborn Infant[Title/Abstract])) OR (Newborn Infants[Title/Abstract])) OR (Neonate[Title/Abstract])) OR (Neonates[Title/Abstract])) OR (Newborns[Title/Abstract])) OR (Newborn[Title/Abstract]) | 301914 |
| #9=#7OR#8 | ("Infant, Newborn"[Mesh]) OR (((((((Infants, Newborn[Title/Abstract]) OR (Newborn Infant[Title/Abstract])) OR (Newborn Infants[Title/Abstract])) OR (Neonate[Title/Abstract])) OR (Neonates[Title/Abstract])) OR (Newborns[Title/Abstract])) OR (Newborn[Title/Abstract])) | 807786 |
| #10=#6OR#9 | (("Infant, Premature"[Mesh]) OR ((((((((Infants, Premature[Title/Abstract]) OR (Premature Infant[Title/Abstract])) OR (Premature Infants[Title/Abstract])) OR (Preterm Infants[Title/Abstract])) OR (Infant, Preterm[Title/Abstract])) OR (Infants, Preterm[Title/Abstract])) OR (Preterm Infant[Title/Abstract])) OR (Neonatal Prematurity[Title/Abstract]))) OR (("Infant, Newborn"[Mesh]) OR (((((((Infants, Newborn[Title/Abstract]) OR (Newborn Infant[Title/Abstract])) OR (Newborn Infants[Title/Abstract])) OR (Neonate[Title/Abstract])) OR (Neonates[Title/Abstract])) OR (Newborns[Title/Abstract])) OR (Newborn[Title/Abstract]))) | 813852 |
| #11 | "Risk Factors"[Mesh] | 1011418 |
| #12 | ((((((((((((((((Factor, Risk[Title/Abstract]) OR (Risk Factor[Title/Abstract])) OR (Population at Risk[Title/Abstract])) OR (Populations at Risk[Title/Abstract])) OR (Risk Scores[Title/Abstract])) OR (Risk Score[Title/Abstract])) OR (Score, Risk[Title/Abstract])) OR (Risk Factor Scores[Title/Abstract])) OR (Risk Factor Score[Title/Abstract])) OR (Score, Risk Factor[Title/Abstract])) OR (Health Correlates[Title/Abstract])) OR (Social Risk Factors[Title/Abstract])) OR (Factor, Social Risk[Title/Abstract])) OR (Factors, Social Risk[Title/Abstract])) OR (Risk Factor, Social[Title/Abstract])) OR (Risk Factors, Social[Title/Abstract])) OR (Social Risk Factor[Title/Abstract]) | 346538 |
| #13=#11OR#12 | ("Risk Factors"[Mesh]) OR (((((((((((((((((Factor, Risk[Title/Abstract]) OR (Risk Factor[Title/Abstract])) OR (Population at Risk[Title/Abstract])) OR (Populations at Risk[Title/Abstract])) OR (Risk Scores[Title/Abstract])) OR (Risk Score[Title/Abstract])) OR (Score, Risk[Title/Abstract])) OR (Risk Factor Scores[Title/Abstract])) OR (Risk Factor Score[Title/Abstract])) OR (Score, Risk Factor[Title/Abstract])) OR (Health Correlates[Title/Abstract])) OR (Social Risk Factors[Title/Abstract])) OR (Factor, Social Risk[Title/Abstract])) OR (Factors, Social Risk[Title/Abstract])) OR (Risk Factor, Social[Title/Abstract])) OR (Risk Factors, Social[Title/Abstract])) OR (Social Risk Factor[Title/Abstract])) | 1217337 |
| #14 | #3AND#10AND#13 | 872 |

**Cochrane library**(347)（2024.11.22）

| Search number | Query | Results |
| --- | --- | --- |
| #1 | Mesh descriptor:[Bronchopulmonary Dysplasia]explode all trees | 788 |
| #2 | (Dysplasia, Bronchopulmonary):ab,ti,kw OR (Chronic lung disease of prematurity):ab,ti,kw | 2293 |
| #3 | #1 OR #2 | 2293 |
| #4 | Mesh descriptor:[Infant,Premature]explode all trees | 5949 |
| #5 | (Infants, Premature):ab,ti,kw OR (Premature Infant):ab,ti,kw OR (Premature Infants):ab,ti,kw OR (Preterm Infants):ab,ti,kw OR (Infant, Preterm):ab,ti,kw OR (Infants, Preterm):ab,ti,kw OR (Preterm Infant):ab,ti,kw OR (Neonatal Prematurity):ab,ti,kw OR (Prematurity, Neonatal):ab,ti,kw | 18383 |
| #6 | #4 OR #5 | 18383 |
| #7 | Mesh descriptor:[Infant,Newborn]explode all trees | 24460 |
| #8 | (Infants, Newborn ):ab,ti,kw OR (Newborn Infant ):ab,ti,kw OR (Newborn Infants ):ab,ti,kw OR (Neonate ):ab,ti,kw OR (Neonates ):ab,ti,kw OR (Newborns ):ab,ti,kw OR (Newborn):ab,ti,kw | 41560 |
| #9 | #7 OR #8 | 41741 |
| #10 | #6 OR #9 | 47358 |
| #11 | Mesh descriptor:[Risk Factors]explode all trees | 38551 |
| #12 | (Factor, Risk):ab,ti,kw OR (Risk Factor):ab,ti,kw OR (Population at Risk):ab,ti,kw OR (Populations at Risk):ab,ti,kw OR (Risk Scores):ab,ti,kw OR (Risk Score):ab,ti,kw OR (Score, Risk):ab,ti,kw OR (Risk Factor Scores):ab,ti,kw OR (Risk Factor Score):ab,ti,kw OR (Score, Risk Factor):ab,ti,kw OR (Health Correlates):ab,ti,kw OR (Social Risk Factors):ab,ti,kw OR (Factor, Social Risk):ab,ti,kw OR (Factors, Social Risk):ab,ti,kw | 143258 |
| #13 | #11 OR #12 | 161798 |
| #14 | #3 AND #10 AND #13 | 347 |

**Embase**(2997)（2024.11.22）

| Search number | Query | Results |
| --- | --- | --- |
| #1 | 'lung dysplasia'/exp | 17253 |
| #2 | 'bronchopulmonary dysplasia':ab,ti OR 'dysplasia, bronchopulmonary':ab,ti OR 'chronic lung disease of prematurity':ab,ti | 13493 |
| #3 | #1 OR #2 | 19225 |
| #4 | 'prematurity'/exp | 147831 |
| #5 | 'infant, premature':ab,ti OR 'infants, premature':ab,ti OR 'premature infant':ab,ti OR 'premature infants':ab,ti OR 'preterm infants':ab,ti OR 'infant, preterm':ab,ti OR 'infants, preterm':ab,ti OR 'preterm infant':ab,ti OR 'neonatal prematurity':ab,ti OR 'prematurity, neonatal':ab,ti | 68111 |
| #6 | #4 OR #5 | 160702 |
| #7 | 'infant, newborn'/exp | 703985 |
| #8 | 'infants, newborn':ab,ti OR 'newborn infants':ab,ti OR 'newborn infant':ab,ti OR 'neonate':ab,ti OR 'neonates':ab,ti OR 'newborn':ab,ti OR 'newborns':ab,ti | 373842 |
| #9 | #7 OR #8 | 827403 |
| #10 | #6 OR #9 | 903624 |
| #11 | 'risk factor'/exp | 1481920 |
| #12 | 'risk factors':ab,ti OR 'factor, risk':ab,ti OR 'population at risk':ab,ti OR 'populations at risk':ab,ti OR 'risk scores':ab,ti OR 'risk score':ab,ti OR 'score, risk':ab,ti OR 'risk factor scores':ab,ti OR 'risk factor score':ab,ti OR 'score, risk factor':ab,ti OR 'health correlates':ab,ti OR 'social risk factors':ab,ti OR 'factor, social risk':ab,ti OR 'factors, social risk':ab,ti OR 'risk factor, social':ab,ti OR 'risk factors, social':ab,ti OR 'social risk factor':ab,ti | 1704711 |
| #13 | #11 OR #12 | 2429074 |
| #14 | #3 AND #10 AND #13 | 2997 |

**Web of science**(2484)（2024.11.22）

| Search number | Query | Results |
| --- | --- | --- |
| #1 | TS=(Bronchopulmonary Dysplasia OR Bronchopulmonary Dysplasia OR Bronchopulmonary Dysplasia) | 13884 |
| #2 | TS=(Risk Factors OR Factor, Risk OR Risk Factor OR Population at Risk OR Populations at Risk OR Risk Scores OR Risk Score OR Score, Risk OR Risk Factor Scores OR Risk Factor Score OR Score, Risk Factor OR Health Correlates OR Correlates, Health OR Social Risk Factors OR Factor, Social Risk OR Factors, Social Risk OR Risk Factor, Social OR Risk Factors, Social OR Social Risk Factor) | 2344088 |
| #3 | TS=((Infant, Premature OR Infants, Premature OR Premature Infant OR Premature Infants OR Preterm Infants OR Infant, Preterm OR Infants, Preterm OR Preterm Infant OR Neonatal Prematurity OR Prematurity, Neonatal OR Infant, Newborn OR Infants, Newborn OR Newborn Infant OR Newborn Infants OR Neonate OR Neonates OR Newborns OR Newborn) ) | 368094 |
| #4 | #1 AND #2 AND #3 | 2484 |
