# Supplementary material for: Risk factors for bronchopulmonary dysplasia in preterm infants: a systematic review and meta-analysis
Source: PeerJ. 2025 Oct 10;13:e20202. doi: 10.7717/peerj.20202 (PMC12517283; doi:10.7717/peerj.20202)
Supplement: Supplemental Information 2 — Quality assessment of included studies using the Newcastle-Ottawa Scale (NOS) for cohort studies and case-control studies. The evaluation covers three key domains: (1) selection bias (representativeness of exposed cohort, selection of non-exposed cohort, etc.), (2) comparability (control for confounding factors), and (3) outcome assessment (assessment method and follow-up adequacy). Results are categorized as low, moderate, or high risk of bias. [file peerj-13-20202-s002.docx]

**The Newcastle-Ottawa Scale (NOS) for Case-control studies**

| **References** | **Selection** | | | | **Comparability** | **exposure** | | | **Total score** |
| --- | --- | --- | --- | --- | --- | --- | --- | --- | --- |
|  | **(1)** | **(2)** | **(3)** | **(4)** | **(5)** | **(6)** | **(7)** | **(8)** |  |
| *(*[*Benali et al. 2024*](#_ENREF_3)*)* | 1 | 1 | 0 | 1 | 0 | 0 | 1 | 1 | 5 |
| *(*[*Dou et al. 2023*](#_ENREF_7)*)* | 1 | 1 | 0 | 1 | 1 | 1 | 1 | 1 | 7 |
| *(*[*Cokyama & Kavuncuoglu 2020*](#_ENREF_5)*)* | 1 | 1 | 0 | 1 | 0 | 1 | 1 | 1 | 6 |
| *(*[*Rojas et al. 2012*](#_ENREF_20)*)* | 1 | 1 | 0 | 1 | 0 | 1 | 1 | 1 | 6 |
| *(*[*Demirel et al. 2009*](#_ENREF_6)*)* | 1 | 1 | 0 | 1 | 0 | 1 | 1 | 1 | 6 |

- 1. Is the case definition adequate?
  2. Representativeness of the cases
  3. Selection of Controls
  4. Definition of Controls
  5. Comparability of cases and controls on the basis of the design or analysis
  6. Ascertainment of exposure
  7. Same method of ascertainment for cases and controls
  8. Non-Response rate

**The Newcastle-Ottawa Scale (NOS) for Cohort studies**

| **References** | **Selection** | | | | **Comparability** | **Outcome** | | | **Total score** |
| --- | --- | --- | --- | --- | --- | --- | --- | --- | --- |
|  | **(1)** | **(2)** | **(3)** | **(4)** | **(5)** | **(6)** | **(7)** | **(8)** |  |
| *(*[*Abushahin et al. 2024*](#_ENREF_1)*)* | 1 | 1 | 1 | 1 | 0 | 1 | 1 | 1 | 7 |
| *(*[*Bolat et al. 2024*](#_ENREF_4)*)* | 1 | 1 | 1 | 1 | 0 | 1 | 1 | 1 | 7 |
| *(*[*Gobec et al. 2023*](#_ENREF_10)*)* | 1 | 1 | 0 | 1 | 0 | 1 | 1 | 1 | 6 |
| *(*[*Huang et al. 2023*](#_ENREF_12)*)* | 1 | 1 | 1 | 1 | 0 | 1 | 1 | 1 | 7 |
| *(*[*Alonso et al. 2022*](#_ENREF_2)*)* | 1 | 1 | 1 | 1 | 0 | 1 | 1 | 1 | 7 |
| *(*[*Ebrahimi et al. 2021*](#_ENREF_9)*)* | 1 | 1 | 1 | 1 | 0 | 1 | 1 | 0 | 6 |
| *(*[*Nakashima et al. 2021*](#_ENREF_14)*)* | 1 | 1 | 0 | 1 | 0 | 1 | 1 | 1 | 6 |
| *(*[*Park et al. 2021*](#_ENREF_16)*)* | 1 | 1 | 1 | 1 | 0 | 1 | 1 | 0 | 6 |
| *(*[*Shin et al. 2020*](#_ENREF_22)*)* | 1 | 1 | 1 | 1 | 0 | 1 | 1 | 1 | 7 |
| *(*[*Patel et al. 2019*](#_ENREF_17)*)* | 1 | 1 | 1 | 1 | 0 | 1 | 1 | 1 | 7 |
| *(*[*Rocha et al. 2019a*](#_ENREF_18)*)* | 1 | 1 | 1 | 1 | 0 | 1 | 1 | 0 | 6 |
| *(*[*Rutkowska et al. 2019*](#_ENREF_21)*)* | 1 | 1 | 1 | 1 | 0 | 1 | 1 | 0 | 6 |
| *(*[*Rocha et al. 2019b*](#_ENREF_19)*)* | 1 | 1 | 1 | 1 | 0 | 1 | 1 | 0 | 6 |
| *(*[*Jung & Lee 2019*](#_ENREF_13)*)* | 1 | 1 | 1 | 1 | 0 | 1 | 1 | 1 | 7 |
| *(*[*Duan et al. 2016*](#_ENREF_8)*)* | 1 | 1 | 1 | 1 | 0 | 1 | 1 | 1 | 7 |
| *(*[*Zhang et al. 2014*](#_ENREF_23)*)* | 1 | 1 | 1 | 1 | 0 | 1 | 1 | 1 | 7 |
| *(*[*Ozkan et al. 2012*](#_ENREF_15)*)* | 1 | 1 | 1 | 1 | 0 | 1 | 1 | 1 | 7 |
| *(*[*Guimarães et al. 2010*](#_ENREF_11)*)* | 1 | 1 | 1 | 1 | 0 | 1 | 1 | 1 | 7 |

1. Representativeness of the exposed cohort
2. Selection of the non-exposed cohort
3. Ascertainment of exposure
4. Demonstration that outcome of interest was not present at start of study
5. Comparability of cohorts on the basis of the design or analysis
6. Assessment of outcome
7. Was follow-up long enough for outcomes to occur
8. Adequacy of follow up of cohorts
