# Supplementary figures and images for: Risk factors for bronchopulmonary dysplasia in preterm infants: a systematic review and meta-analysis
Source: PeerJ. 2025 Oct 10;13:e20202. doi: 10.7717/peerj.20202 (PMC12517283; doi:10.7717/peerj.20202)

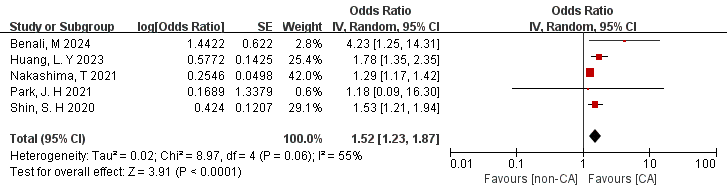

Supplement: Supplemental Information 3 — Forest plot of chorioamnionitis (CA) as a risk factor for bronchopulmonary dysplasia (BPD) using random-effects model (pooled OR=1.52, 95% CI 1.23-1.87, I²=55%) [file peerj-13-20202-s003.png]

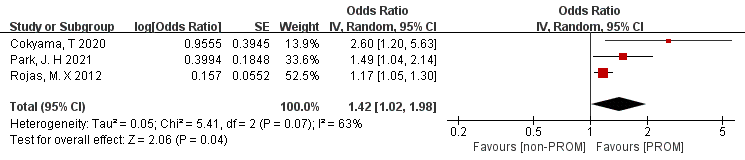

Supplement: Supplemental Information 4 — Forest plot of premature rupture of membranes (PROM) as a risk factor for bronchopulmonary dysplasia (BPD) using random-effects model (pooled OR=1.42, 95% CI 1.02-1.98, I²=63%) [file peerj-13-20202-s004.png]

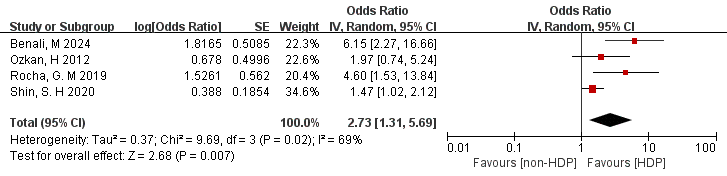

Supplement: Supplemental Information 5 — Forest plot of hypertensive disorders pregnancy (HDP) as a risk factor for bronchopulmonary dysplasia (BPD) using random-effects model (pooled OR=2.73, 95% CI 1.31-5.69, I²=69%) [file peerj-13-20202-s005.png]

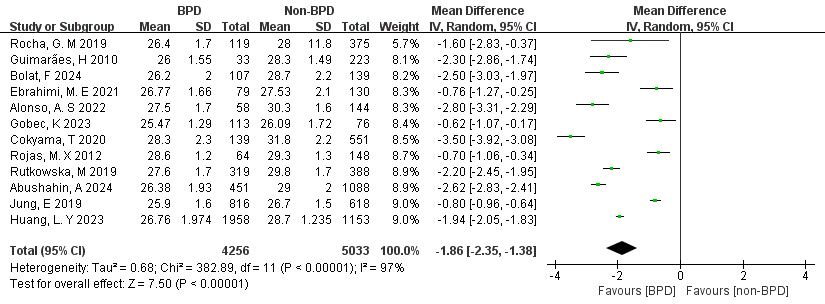

Supplement: Supplemental Information 6 — Forest plot of gestational age (GA) as a risk factor for bronchopulmonary dysplasia (BPD) using random-effects model (pooled MD=-1.86, 95% CI -2.35, -1.38, I²=97%) [file peerj-13-20202-s006.png]

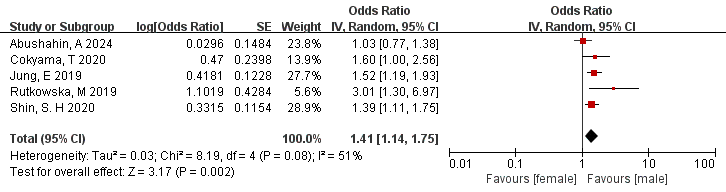

Supplement: Supplemental Information 7 — Forest plot of sex as a risk factor for bronchopulmonary dysplasia (BPD) using random-effects model (pooled OR=1.41, 95% CI 1.14-1.75, I²=51%) [file peerj-13-20202-s007.png]

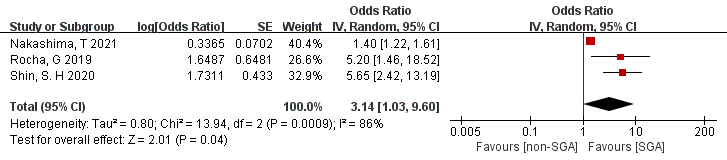

Supplement: Supplemental Information 8 — Forest plot of small for gestational age (SGA) as a risk factor for bronchopulmonary dysplasia (BPD) using random-effects model (pooled OR=3.14, 95% CI 1.03-9.60, I²=86%) [file peerj-13-20202-s008.png]

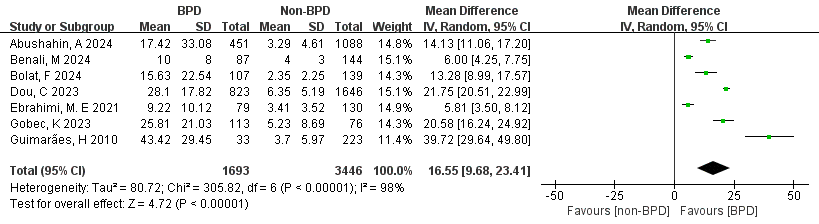

Supplement: Supplemental Information 9 — Forest plot of mechanical ventilation ( MV) as a risk factor for bronchopulmonary dysplasia (BPD) using random-effects model (pooled MD=16.55, 95% CI 9.68-23.41, I²=98%) [file peerj-13-20202-s009.png]

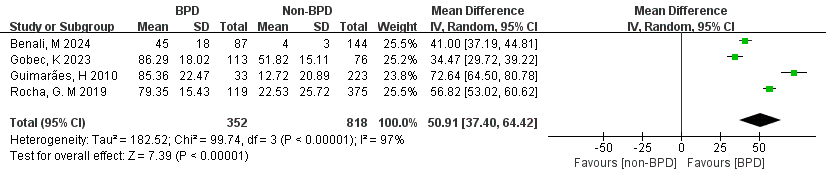

Supplement: Supplemental Information 10 — Forest plot of oxygen adnimistration as a risk factor for bronchopulmonary dysplasia (BPD) using random-effects model (pooled MD=50.91, 95% CI 37.40-64.42, I²=97%) [file peerj-13-20202-s010.png]

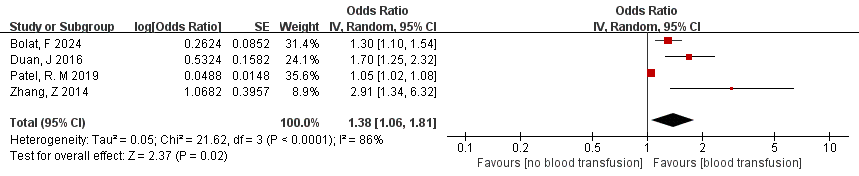

Supplement: Supplemental Information 11 — Forest plot of blood transfusion as a risk factor for bronchopulmonary dysplasia (BPD) using random-effects model (pooled OR=1.38, 95% CI 1.06-1.81, I²=86%) [file peerj-13-20202-s011.png]

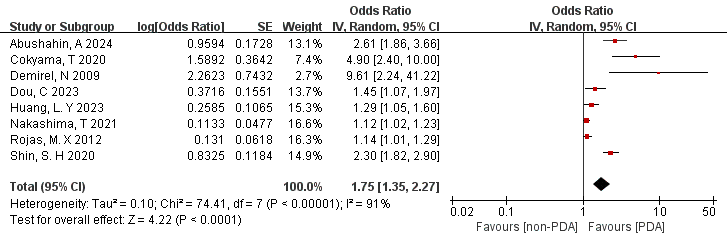

Supplement: Supplemental Information 12 — Forest plot of patent ductus arteriosus (PDA) as a risk factor for bronchopulmonary dysplasia (BPD) using random-effects model (pooled OR=1.75, 95% CI 1.35-2.27, I²=91%) [file peerj-13-20202-s012.png]

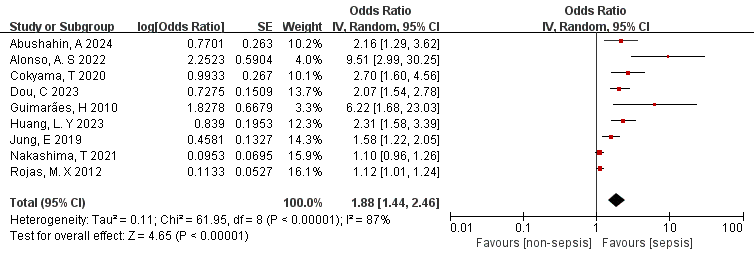

Supplement: Supplemental Information 13 — Forest plot of sepsis as a risk factor for bronchopulmonary dysplasia (BPD) using random-effects model (pooled OR=1.88, 95% CI 1.44-2.46, I²=87%) [file peerj-13-20202-s013.png]

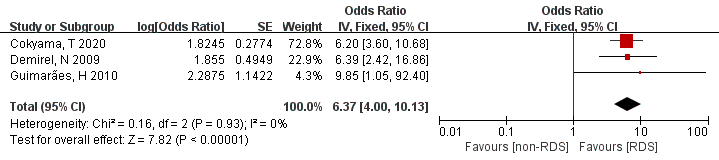

Supplement: Supplemental Information 14 — Forest plot of r espiratory distress syndrome (RDS) as a risk factor for bronchopulmonary dysplasia (BPD) using fixed-effects model (pooled OR=6.37, 95% CI 4.0-10.13, I²=0%) [file peerj-13-20202-s014.png]

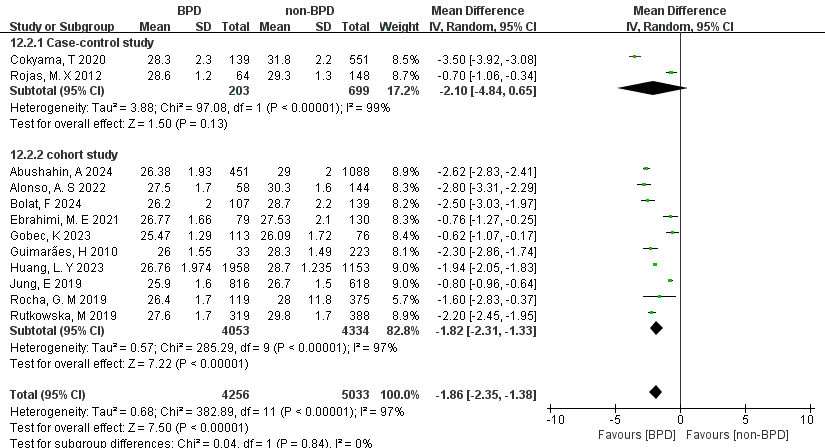

Supplement: Supplemental Information 15 — Forest plot of gestational age (GA) as a risk factor for bronchopulmonary dysplasia (BPD), stratified by study design using random-effects model. Mean differences (MD) with 95% confidence intervals are presented separately for cohort (MD= -1.82 weeks, 95% CI -2.31,-1.33) and case-control studies (MD= -2.10 weeks, 95% CI -4.84, 0.65). Heterogeneity was quantified by I² statistics (cohort: I²=97%; case-control: I²=99%). Test for subgroup differences: p=0.84. [file peerj-13-20202-s015.png]

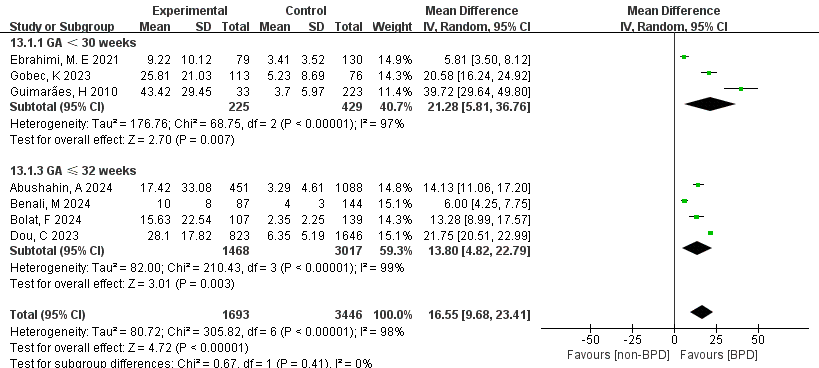

Supplement: Supplemental Information 16 — Different GA stratifications on MV duration through subgroup analysis. In the very preterm infant subgroup (GA <30 weeks), the pooled results demonstrated a significantly prolonged MV duration (MD=21.28 days, 95% CI 5.81-36.76), albeit with extremely high heterogeneity (I²=97%). Similarly, in the very preterm infant subgroup (GA ≤32 weeks), MV duration was also significantly extended (MD=13.80 days, 95% CI 4.82-22.79), with even higher heterogeneity (I²=99%). T he test for subgroup differences showed no statistical significance (P=0.41, I²=0%). [file peerj-13-20202-s016.png]

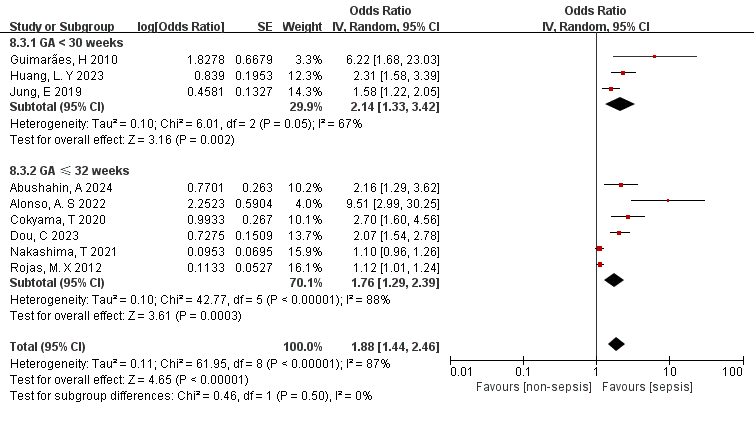

Supplement: Supplemental Information 17 — Random-effects model forest plot of sepsis as a risk factor for BPD, stratified by gestational age groups (<30 weeks, ≤32 weeks). Pooled odds ratios (ORs) with 95% confidence intervals are shown for each stratum. Heterogeneity quantified by I² statistics.Test for subgroup differences: p=0.50. [file peerj-13-20202-s017.png]

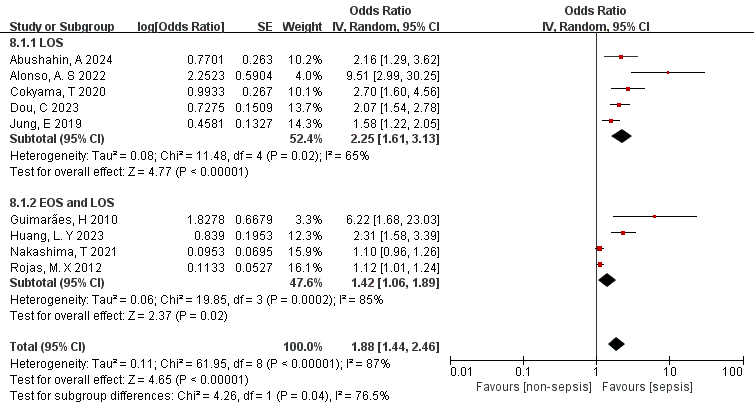

Supplement: Supplemental Information 18 — Forest plot demonstrating the association between neonatal sepsis (stratified by diagnostic criteria: late-onset sepsis (LOS) vs LOS / early-onset sepsis (EOS) ) and bronchopulmonary dysplasia (BPD) using random-effects meta-analysis. Pooled odds ratios (ORs) with 95% confidence intervals are displayed for each subgroup. Heterogeneity was assessed using I² statistics. Test for subgroup differences: p=0.04. [file peerj-13-20202-s018.png]

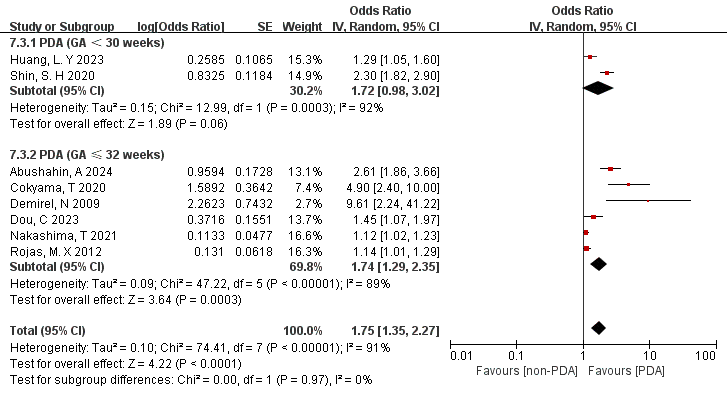

Supplement: Supplemental Information 19 — Random-effects model forest plot of patent ductus arteriosus (PDA) as a risk factor for BPD, stratified by gestational age groups (<30 weeks, ≤32 weeks ). Pooled odds ratios (ORs) with 95% confidence intervals are shown for each stratum. Heterogeneity quantified by I² statistics.Test for subgroup differences: p=0.97. [file peerj-13-20202-s019.png]

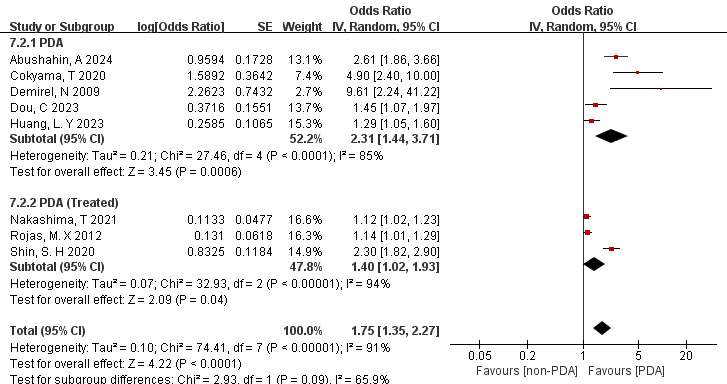

Supplement: Supplemental Information 20 — Forest plot of PDA-associated BPD risk stratified by treatment exposure (treated [pharmacologic/surgical] vs untreated). Pooled odds ratios (OR) with 95% CIs derived from random-effects models. Heterogeneity: treated (I²=94%), untreated (I²=85%). Subgroup difference p=0.09. [file peerj-13-20202-s020.png]

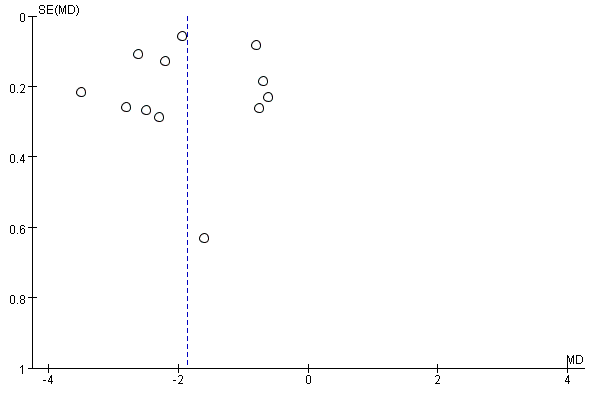

Supplement: Supplemental Information 22 — Each point represents an individual study’s effect size (MD in weeks) against its precision (1/SE). Asymmetry was quantified using Egger’s test (p=0.789). [file peerj-13-20202-s022.png]
