# Supplementary material for: Risk factors for bronchopulmonary dysplasia in preterm infants: a systematic review and meta-analysis
Source: PeerJ. 2025 Oct 10;13:e20202. doi: 10.7717/peerj.20202 (PMC12517283; doi:10.7717/peerj.20202)
Supplement: Supplemental Information 21 — Sensitivity analysis evaluating the robustness of all identified risk factors for bronchopulmonary dysplasia (BPD). Effect estimates (odds ratios or m ean difference ) were recalculated by sequentially excluding individual studies. [file peerj-13-20202-s021.pdf]

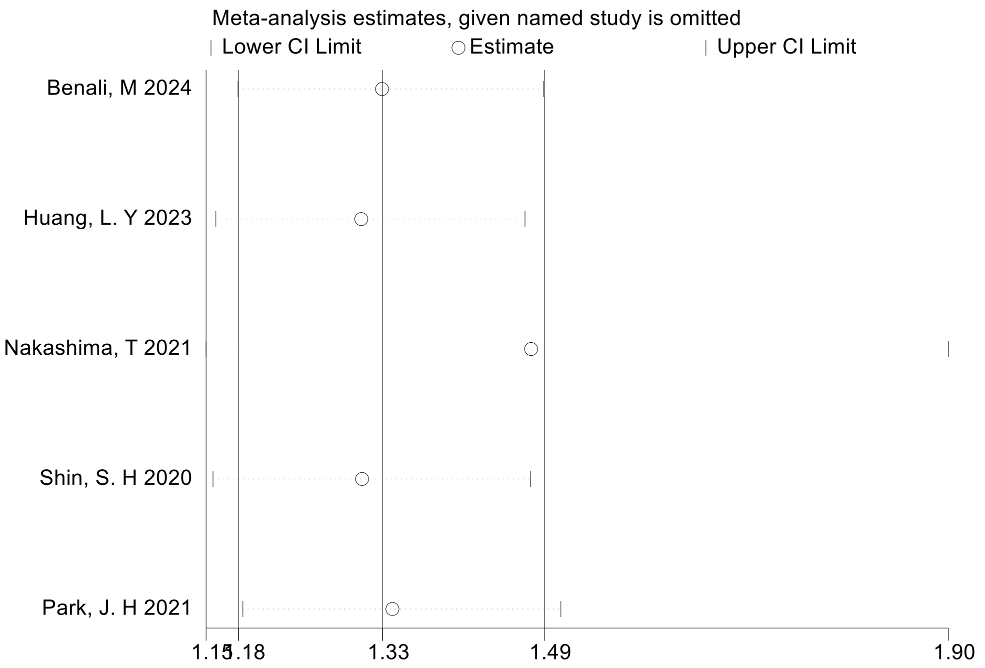

Figure A. Sensitivity analysis plot for CA

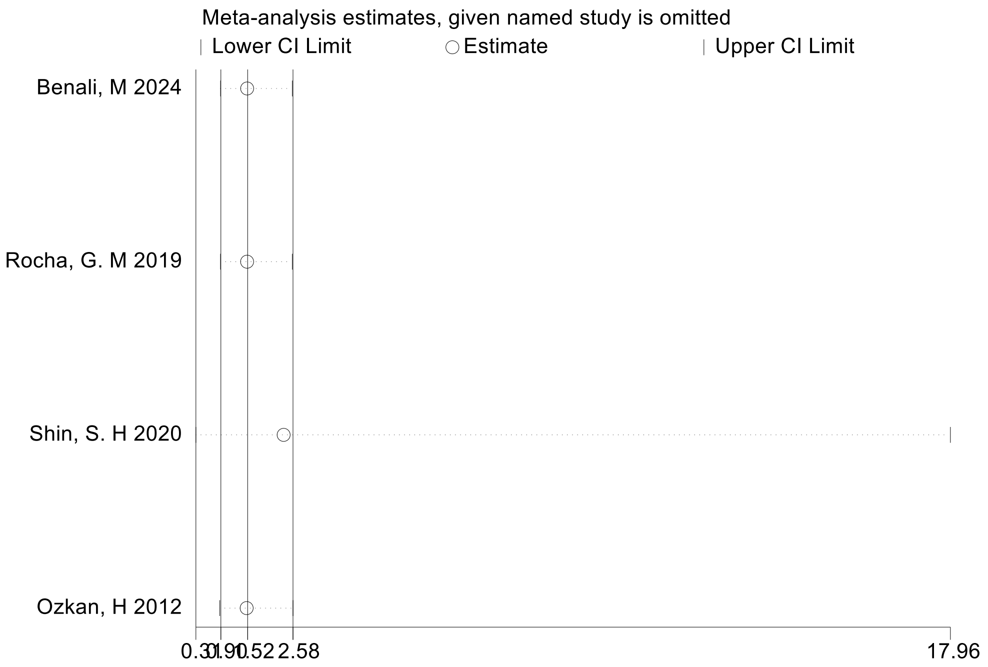

Figure B. Sensitivity analysis plot for HDP

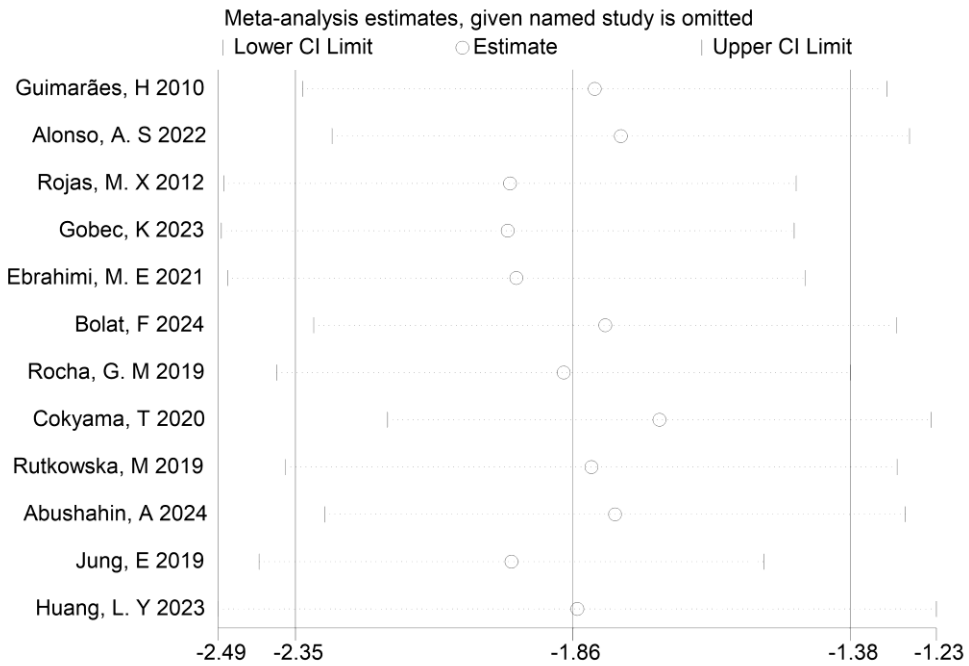

Figure C. Sensitivity analysis plot for GA

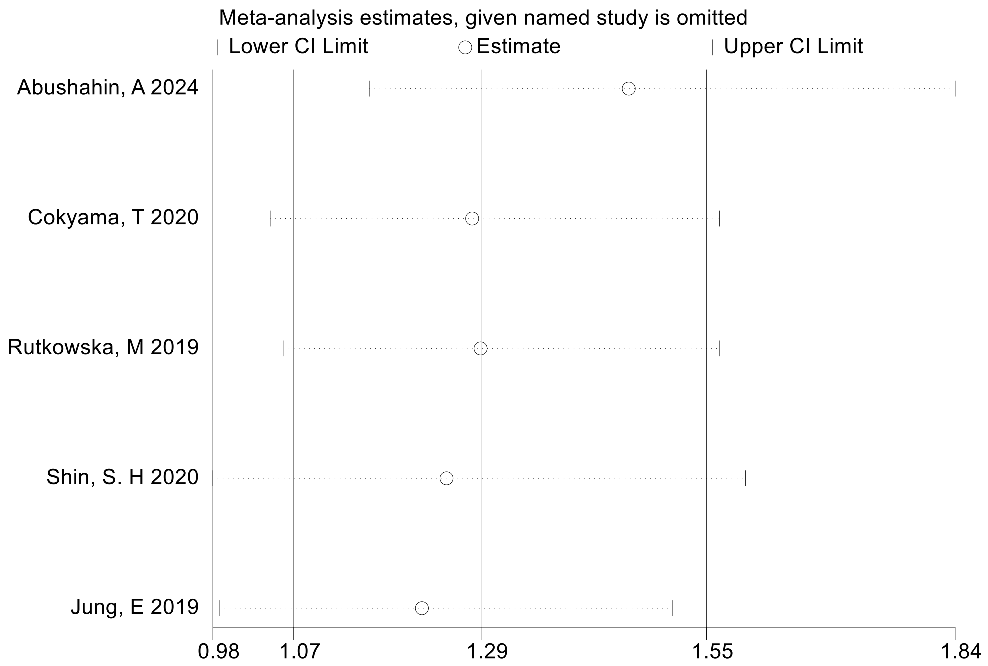

Figure D. Sensitivity analysis plot for male

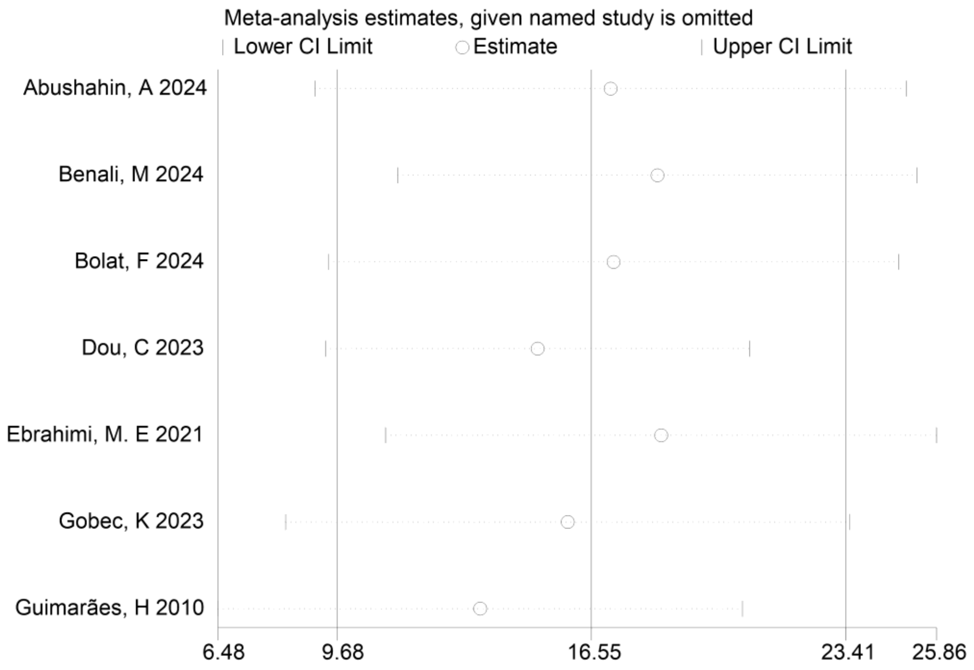

Figure E. Sensitivity analysis plot for MV

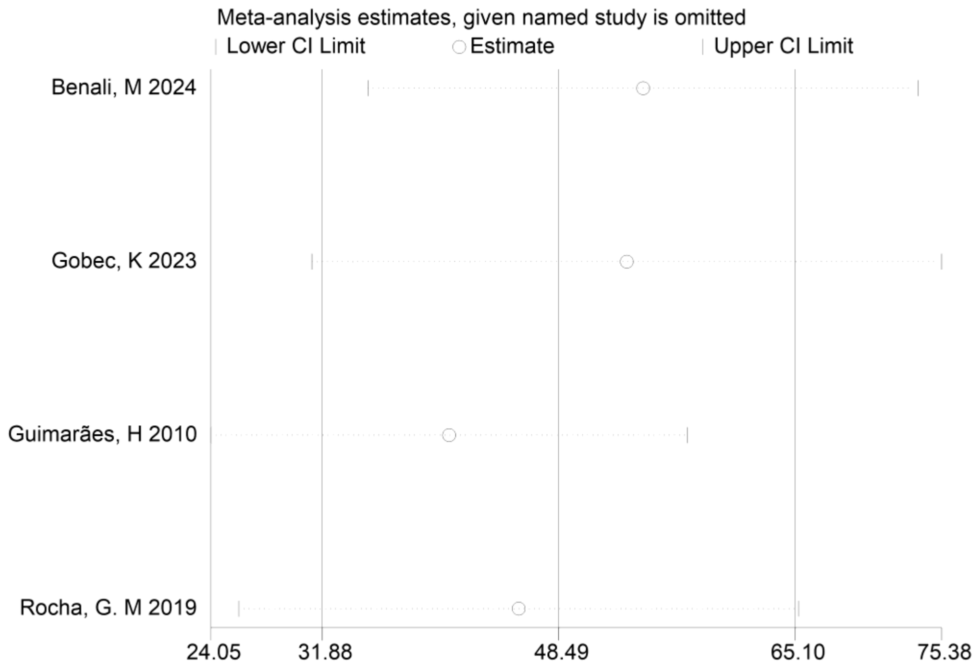

Figure F. Sensitivity analysis plot for oxygen administration

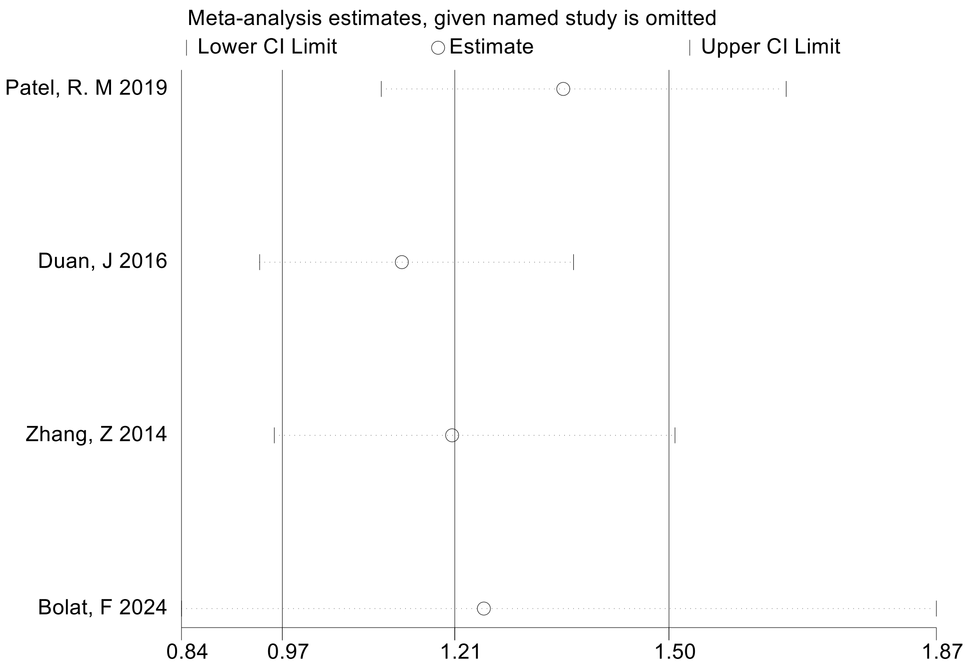

Figure G. Sensitivity analysis plot for blood transfusion

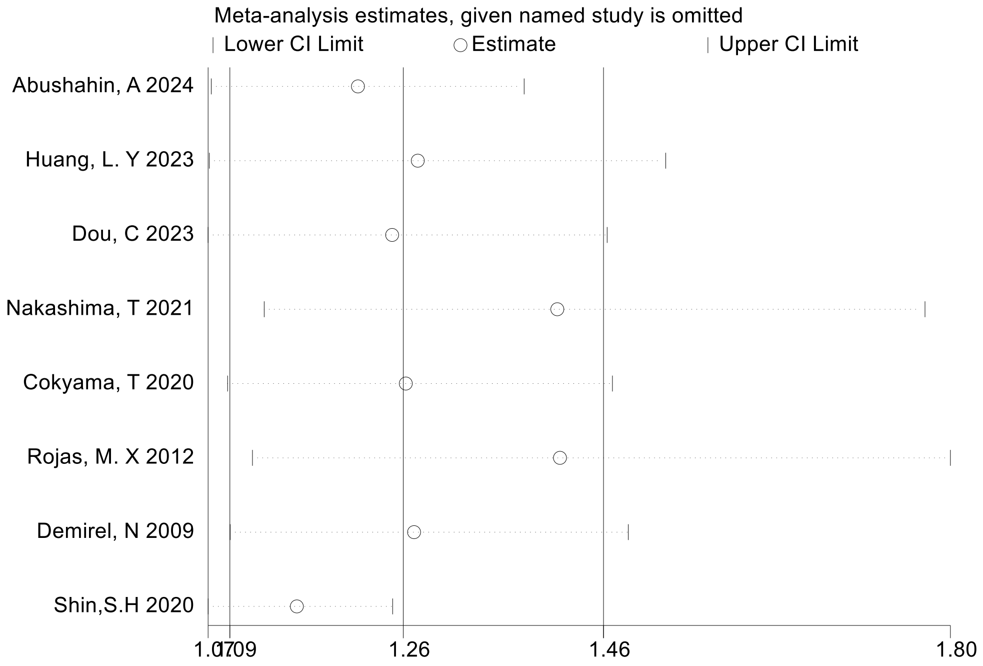

Figure H. Sensitivity analysis plot for PDA

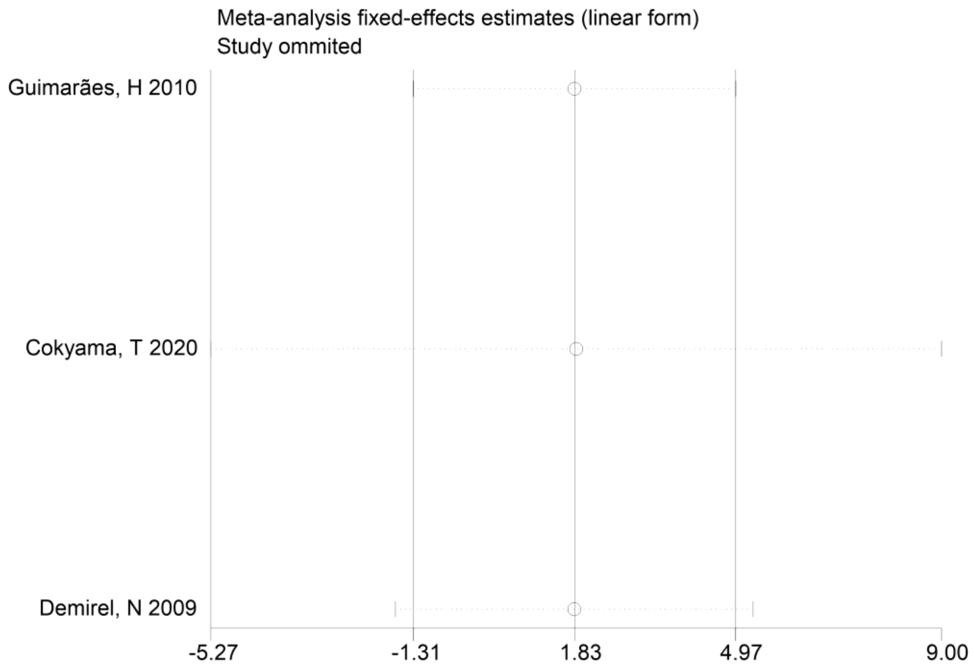

Figure I. Sensitivity analysis plot for RDS

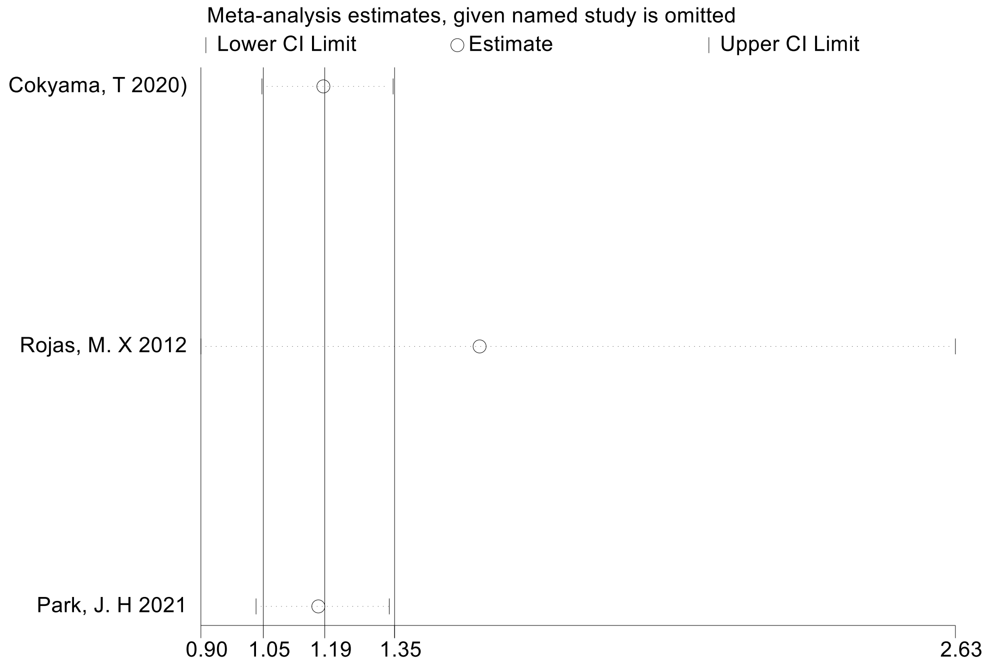

Figure J. Sensitivity analysis plot for PROM

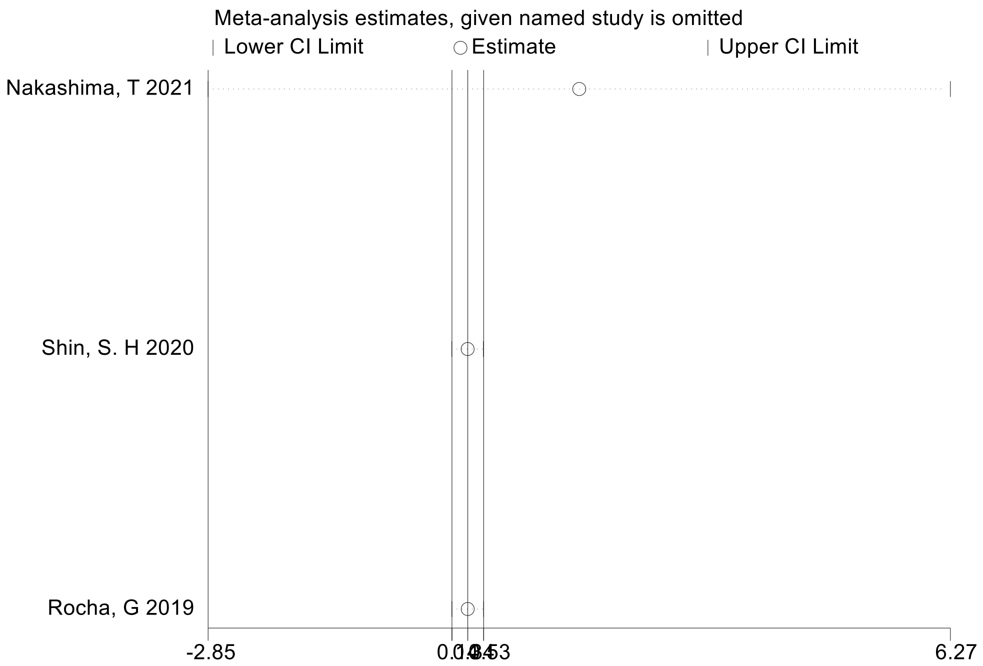

Figure K. Sensitivity analysis plot for SGA

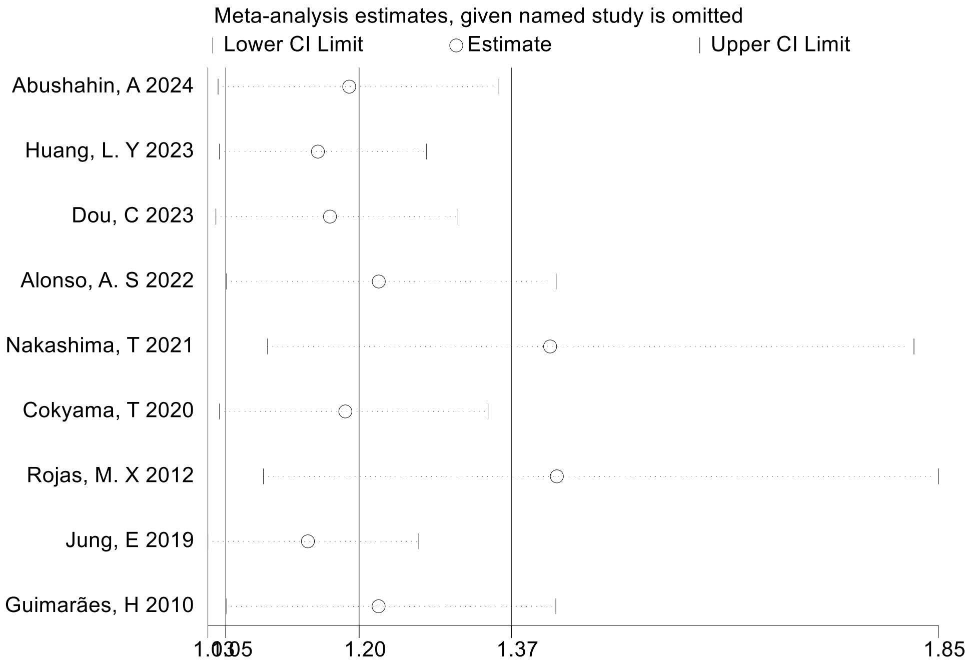

Figure L. Sensitivity analysis plot for sepsis
