# Supplementary material for: Risk factors for bronchopulmonary dysplasia in preterm infants: a systematic review and meta-analysis
Source: PeerJ. 2025 Oct 10;13:e20202. doi: 10.7717/peerj.20202 (PMC12517283; doi:10.7717/peerj.20202)
Supplement: Supplemental Information 23 — With the development of medical technology, more and more premature babies are surviving, but bronchopulmonary dysplasia (BPD) has a high prevalence in the very premature population, and these children with BPD have a long initial hospital stay, a high frequency of hospital readmissions, and a high percentage of subsequent admissions to the pediatric intensive care unit for lung disease, which may even be secondary to pulmonary arterial hypertension. Treatment of BPD is limited and there are no safe and effective medications. Therefore, prevention is more important, and knowledge of risk factors is the basis for identifying preventive measures. This is why a meta-analysis is needed to summarize the relevant risk factors. We conducted a comprehensive, evidence-based study on the risk factors for BPD in preterm infants, evaluating a total of 12 potential risk factors, including three different time periods: prenatal, intrapartum and postnatal. [file peerj-13-20202-s023.docx]

**The rationale for conducting the meta-analysis**

With the development of medical technology, more and more premature babies are surviving, but bronchopulmonary dysplasia (BPD) has a high prevalence in the very premature population, and these children with BPD have a long initial hospital stay, a high frequency of hospital readmissions, and a high percentage of subsequent admissions to the pediatric intensive care unit for lung disease, which may even be secondary to pulmonary arterial hypertension. Treatment of BPD is limited and there are no safe and effective medications. Therefore, prevention is more important, and knowledge of risk factors is the basis for identifying preventive measures. This is why a meta-analysis is needed to summarize the relevant risk factors.

**The contribution that the meta-analysis makes to knowledge in light of previously published related reports, including other meta-analyses and systematic reviews.**

In extant literature on the subject, the preponderance of reports has hitherto focused on the evaluation of individual risk factors, including sepsis, patent ductus arteriosus, and chorioamnionitis, among others. The challenge lies in formulating a comprehensive demonstration of the risk factors associated with BPD.

In this study, we conducted a comprehensive, evidence-based study on the risk factors for BPD in preterm infants, evaluating a total of 12 potential risk factors, including three different time periods: prenatal, intrapartum and postnatal.

It is hoped that the study will enable clinicians to recognize that the risk factors for BPD have a different focus over time. The study's findings may serve as a crucial impetus for clinicians to re-evaluate their practices and inform clinical decision-making.
